# Trends in Consumption of Ultra-Processed Foods Among Adults in Southern China: Analysis of Serial Cross-Sectional Health Survey Data 2002–2022

**DOI:** 10.3390/nu16234008

**Published:** 2024-11-23

**Authors:** Shiqi Li, Jingtai Ma, Jian Wen, Jiewen Peng, Panpan Huang, Lilian Zeng, Siyi Chen, Guiyuan Ji, Xingfen Yang, Wei Wu

**Affiliations:** 1 NMPA Key Laboratory for Safety Evaluation of Cosmetics, Guangdong Provincial Key Laboratory of Tropical Disease Research, School of Public Health, Southern Medical University, Guangzhou 510515, China; lishiqi1999@126.com (S.L.); jingtaima@163.com (J.M.); 2 Guangdong Provincial Institute of Public Health, Guangdong Provincial Center for Disease Control and Prevention, Guangzhou 511400, China; gdpiph2017@163.com (J.P.); zenglilian@yeah.net (L.Z.); chensiyi1985@126.com (S.C.); 3 Guangdong Provincial Center for Disease Control and Prevention, Guangzhou 511400, China; wenjian19750726@126.com (J.W.); sjkzx_sas@gd.gov.cn (P.H.)

**Keywords:** ultra-processed foods, consumption, nutrient, adults, China National Nutrition Surveys

## Abstract

Background: Over time, there have been significant changes in the dietary patterns of the Chinese population with the emergence of ultra-processed foods (UPFs). Methods: To ascertain the changes in UPFs intake among adults in southern China, over the past two decades, the study included residents aged 18 years and older who participated in the China National Nutrition Surveys in Guangdong province in 2002, 2012, and 2022. Dietary intake data were collected via three-day 24 h dietary recalls and weighing household foods and condiments. The recorded dietary data were classified according to the NOVA classification system, and the percentage of total energy derived from each food group was calculated. Results: From 2002 to 2022, there was a notable increase in the consumption of UPFs among adults in Guangdong Province, with the percentage of UPFs intake rising from 0.88% to 8.52% (*p*-value < 0.001). This growth was especially pronounced among specific population groups, including the young, the better educated, and those living in highly urbanized areas. The largest increase in energy intake from UPFs was observed among students, from 0.42% to 17.26% (*p*-value < 0.001). The nutrient contents of UPFs in Guangdong in 2022 were found to contain a markedly higher calculated percentage of calories provided by carbohydrates in comparison to minimally processed foods (56.6% vs. 43.8%) as well as in sodium (749 mg/100 kcal vs. 29 mg/100 kcal). Conclusions: Given the increasing consumption of UPFs and the growing evidence linking these products to chronic diseases, it is important to promote healthy food intake and balanced diets through active nutritional education campaigns to prevent potential health risks that may arise.

## 1. Introduction

With the accelerated growth of the global economy, there has been a notable shift in the dietary habits of the world’s population towards ultra-processed foods (UPFs). Such foods are defined as industrial formulations, primarily composed of refined or extracted ingredients, which are typically supplemented with a multitude of additives, yet are notably lacking in whole foods [1]. Although the development of food processing has brought about a certain increase in food variety and safety [2,3], the issue lies in the fact that UPFs not only diminish the inherent nutritional value of the food, but also introduce additional amounts of oil, salt, and sugar during their production [4,5], making people consume more energy. Cohort studies consistently show that a high level of consumption of UPFs is a significant contributing factor to the risk of obesity [6,7,8] and cardiovascular diseases [9] risks in adults and is linked to an increased hazard for depression [10,11], as well as all-cause mortality [12,13]. Furthermore, diets comprising a high proportion of UPFs are associated with an increased risk of nutritional imbalance [14,15].

Meanwhile, the structure of the Chinese diet is undergoing major changes. Dietary patterns have gradually shifted from a traditional diet based on grains and vegetables to one associated with high intakes of fat and calorie-dense foods [16]. The consumption of fruits, dairy products, fast food, and drinks rose considerably, while the consumption of rice fell sharply [17]. Dietary preferences are shifting towards high-fat, low-carbohydrate, low-fiber diets [18], coinciding with a shift in the consumption of UPFs.

Guangdong, as a province near to the port, has been at the frontier of economic development and openness since the implementation of the economic reform and open policy. This has created a favorable environment for the food industry to flourish and for foreign goods to enter the Guangdong market. Given the changes in food types and lifestyle transitions that are occurring in Guangdong, it is likely that trends in the intake of processed foods will be seen more clearly than in other regions.

Gaining an understanding of dietary trends is crucial for promoting dietary guidance and nutrition education actions to enhance diet quality and protect the public from diet-related chronic diseases. Equally, it is vital to determine how dietary trends vary for specific socio-demographic subgroups to inform public health efforts to address these differences. There is a lack of long-term dietary surveillance studies on food processing. To address these deficiencies in knowledge, we examined long-term trends in dietary intake by level of processing for different socio-demographic segments of the Guangdong adult population between 2002 and 2022. Further analysis was conducted on the nutrient profiles associated with UPFs consumed in the most recent round of surveys (2022). Such information may serve to guide the formulation of policies and priorities pertaining to the consumption of UPFs, thereby enhancing the dietary quality of adults in Guangdong.

## 2. Materials and Methods

### 2.1. Study Design and Population

The present study was based on three rounds of the China National Nutrition Surveys (CNNS) in 2002, 2012, and 2022. The CNNS represents a nationally surveyed program conducted by the Chinese Centre for Disease Control and Prevention at five to ten-year intervals, beginning in 1959. Multistage stratified cluster sampling methods are employed to select residents of all Chinese provinces. The particulars of the design of the survey and the methods employed have been described elsewhere [19]. We used the data from the Guangdong Province portion of the CNNS survey for analysis.

**Figure 1:**
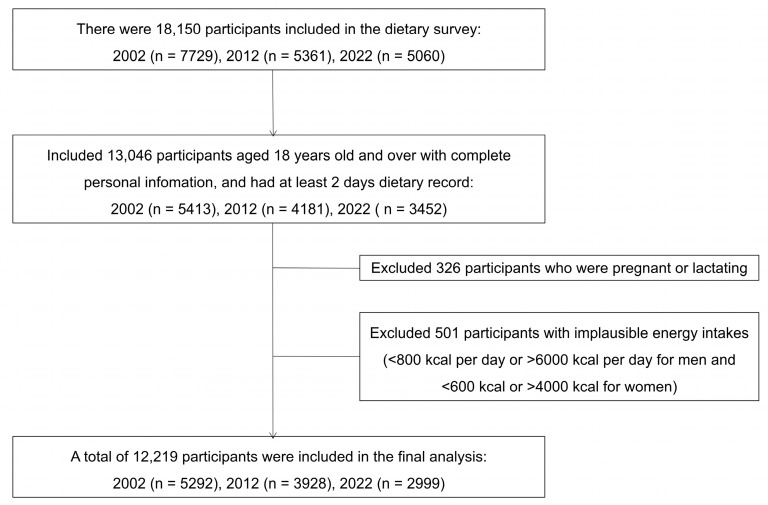
Flowchart of inclusion into the study.

We excluded the subjects according to the following exclusion criteria: (1) participants with age under 18 years, 5104 in total; (2) pregnant women or breastfeeding mothers, 326 in total; (3) participants with implausible energy intakes (<800 kcal/day or >6000 kcal/day for men; <600 kcal/day or >4000 kcal/day for women), 406 in total. Finally, a total of 12,219 adults aged 18 years old or older were included in the present study. The flow of inclusion is shown in Figure 1.

Approval for the series of nationwide surveys was granted by the Ethics Committee of the National Institute of Nutrition and Health, Chinese Center for Disease Control and Prevention (Approval Number: 2022-008, approval date: 28 January 2022). Prior to the commencement of the investigation, all participants were required to sign an informed consent form.

### 2.2. Data Collection

Demographic and socioeconomic information was personally provided by respondents during at-home interviews conducted by trained staff.

To obtain comprehensive dietary data, a combination of 24 h food recall over three consecutive days and a weighing method was applied to assess dietary intake. The previous 24 h dietary intake was documented for each recall day, with two weekdays and one weekend day included. Culinary seasonings were assessed separately, with trained investigators carefully weighing each food item in the household inventory. The Chinese Food Composition Table is used to calculate total daily energy and nutrient intakes [20].

### 2.3. Definition of NOVA Food Groups

In accordance with the NOVA food classification system, food items were categorized into four groups, including minimally processed foods (MPFs) [group 1], culinary seasonings [group 2], processed foods [group 3], and ultra-processed foods (UPFs) [group 4]. These four categories are referred to as the NOVA food groups. MPFs refer to food in its natural form without any alteration, and these foods do not contain added substances such as salt, sugar, or oil. Many MPFs are prepared into dishes at home or in restaurants. Culinary seasonings are substances extracted from nature through processes such as pressing, grinding, crushing, or refining. Processed foods are industrially produced products that may contain preservatives, antioxidants, or stabilizers. Examples are canned vegetables and fruits, canned meat products, cheese, and seasoned nuts.

UPFs are ready-to-eat, ready-to-drink, and ready-to-cook foods. In addition to natural ingredients such as sugar, salt, and oil, UPFs may contain food additives such as flavorings, colorings, emulsifiers, sweeteners, and other additives, and their products often undergo intensive industrial processing such as pre-frying, molding, extrusion, and hydrogenation. In addition to instant noodles, frozen dishes, packaged snacks, and sausages, this food group also includes soy sauce, and chicken essence, which are commonly used in cooking.

For food items that could not be clearly classified, one or more food substances present in the ingredient list that are not used in kitchens, such as fructose and inverted sugar, were identified as UPFs. Two researchers conducted the classification of food items according to the NOVA system separately and a third researcher verified the results. In instances where classification was ambiguous or when disaggregation of recipes (homemade or industrialized) raised doubts, a group discussion was held to determine the appropriate classification. The classification deemed to be the most conservative in nature was then chosen. A detailed description of the NOVA classification can be found in other literature [1].

### 2.4. Population Subgroups

Trends in consumption of UPFs were further assessed among population subgroups by gender, place of residence (urban city and rural areas), age group (18–44 y, 45–59 y, and ≥60 y), education level (primary school or below, secondary school, college or above), occupation (student, retired or unemployed, office worker, business service, manual labor, and others), income (poverty, non-poverty, and no response). The income classification criteria refer to the OECD principles, with a year-specific poverty threshold set by using 50% of the median per capita disposable income for that year [21].

### 2.5. Statistical Analysis

In this study, the proportion of energy intake was selected as the unit of measurement to account for overall energy consumption. This approach minimizes errors in measurement and unrelated fluctuations in dietary patterns, such as those influenced by metabolic rate or physical activity levels. Sampling weights for the post-stratified population in each survey round were derived from 2020 census sampling probabilities, adjusting the standardized data for age and gender distribution.

Categorical variables were expressed as absolute frequencies and percentages, whereas continuous variables were described by medians with interquartile range (IQR). Comparisons of intake between food groups in each year and intake of UPFs between population subgroups in each year were conducted using the Kruskal–Wallis test. Nutrient comparisons were conducted using the Mann–Whitney test. Following the division of the percentage of energy intake from UPFs into four equal parts, the Jonckheere–Terpstra test was utilized to ascertain whether alterations in UPFs intake exhibited a notable trend effect on changes in nutrient content [22].

All analyses were performed using R software (version 4.4.0; R Foundation for Statistical Computing, Vienna, Austria), and a two-sided *p* < 0.05 was considered statistically significant.

## 3. Results

### 3.1. Baseline Characteristics

As presented in Table 1, the total number of adults aged 18 and above included in this analysis was 12,219. Over time, the proportion of those age over 45 years old rose, while the proportion of younger individuals declined. Additionally, there was an increase in the proportion of individuals with higher education, with the percentage of those with a university degree or higher rising from 10.4% in 2002 to 33.2% in 2022. The proportion of non-poverty people was also on the rise, from 52.8% to 61.1%.

### 3.2. Trends in Consumption of NOVA Food Groups and Subgroups

The weighted adjusted median intake of NOVA food groups and subgroups for each survey is detailed in Table 2. From 2002 to 2022, there was a notable increase in the calculated percentage of total energy derived from the consumption of UPFs, rising from 0.88% to 8.52% (*p*-value < 0.001). Conversely, the percentage of total energy derived from the consumption of MPFs experienced a significant decline, decreasing from 82.95% to 68.69% (*p*-value < 0.001). Furthermore, the calculated percentage of energy consumed from culinary seasonings and processed foods also demonstrated a notable shift. Specifically, the calculated percentage of energy derived from processed foods exhibited an increase, while that derived from processed culinary ingredients exhibited a decrease.

Table S2 shows that for the people who have consumed the subgroup UPFs revealed a noteworthy increase in the calculated percentage of energy intake attributed to industrial grain foods, prepared dishes, and other UPFs. A distinct increase was observed in energy intake for industrial grain foods (from 0.53% to 7.15%, *p*-value < 0.001), prepared dishes (from 3.47% to 10.38%, *p*-value < 0.001), and other UPFs (from 0.54% to 0.63%, *p*-value < 0.001) from 2002 to 2022. In contrast, the calculated percentage of energy intake from sugar-sweetened beverages, flavored dairy products, and dairy substitutes differed significantly between surveys from 2002 to 2022 (*p*-value < 0.001) but did not show an increasing trend across surveys. However, there was no significant difference in the calculated percentage of energy intake in the snack and confectionery groups (*p* > 0.05).

### 3.3. Trends in Population Subgroups

Table 3 presents a clear upward tendency in the consumption of UPFs across various population subgroups from 2002 to 2022. The upward trend in UPFs was more pronounced among females, urban dwellers, and younger age groups than among males, rural residents, and older age groups. The consumption of UPFs was found to be greater among adults with a college education or higher, with a notable increase from 0.81% in 2002 to 11.37% in 2022 (*p*-value < 0.001). A comparison of data from 2002 and 2022 revealed that students exhibited the most marked increase (from 0.42% to 17.26%, *p*-value < 0.001), followed by working adults (from 1.04% to 10.22%, *p*-value < 0.001). Irrespective of income bracket, there was a growing prevalence of UPFs consumption, with the most pronounced observed among non-poor households, from 1.05% in 2022 to 10.22% in 2022 (*p*-value < 0.001).

### 3.4. Nutrient Profiles of UPFs

At the level of macronutrients, the UPFs consumed by adults in Guangdong in 2022 were found to contain a markedly higher calculated percentage of calories provided by carbohydrates in comparison to MPFs (56.56% vs. 43.83%), while the percentage of calories from protein (18.36% vs. 22.23%) and total fat (25.08% vs. 33.67%) was observed to be lower. For micronutrients, the sodium content was significantly elevated in UPFs in comparison to MPFs (749.33 mg/100 kcal vs. 29.31 mg/100 kcal). Similarly, the iron levels were also higher in UPFs. However, other micronutrients, including vitamins A, C, and E, carotenoids, calcium, potassium, selenium, phosphorus, magnesium, and zinc, exhibited lower concentrations in UPFs than in MPFs (Table 4).

Table 5 shows the nutrients across quintiles of the UPFs dietary share. For macronutrients, the energy ratios of carbohydrates and proteins exhibited a decline as the intake of UPFs increased, while the energy ratio of fats tended to increase (*p*-value < 0.001). For micronutrients, the consumption of UPFs increased, the density of vitamins A, C, E, and carotene increased, whereas the densities of calcium, potassium, sodium, iron, selenium, phosphorus, magnesium, and zinc decreased significantly (*p*-value < 0.001).

## 4. Discussion

The current research provides a better understanding of processed food consumption among adults in Guangdong Province, as well as the differences in consumption among population groups with potential dietary variations. It can be observed that from 2002 to 2022, there was an increase in the consumption of UPFs among adults in Guangdong Province, with ready-to-eat foods showing the largest increase, while the intake of snacks and desserts decreased. At the same time, the consumption of unprocessed foods has declined across all groups, primarily reflected in the reduced consumption of cereal-based foods. Notably, similar increases in consumption of UPFs have been observed in several regions worldwide [23,24].

It is noteworthy that, within the context of the overall increase, the increase was particularly pronounced among young people, people with higher levels of education or people living in highly urbanized areas, which is similar to the findings of previous studies [25]. People who live in more urbanized areas, or who have a higher level of education, income and socio-economic status, tend to have a higher intake of UPFs compared to the general population, as they have easier access to UPFs [25,26,27]. Changes in age may have an impact on the physiological or psychological regulation of food intake and thus on food choices [28,29]. The finding in the study that younger adults consume a greater amount of UPFs may be attributed to the fact that younger adults tend to consume food prepared outside the home and at work, and engage in less cooking, whereas older adults spend more time cooking and are less familiar with convenience foods [30,31,32]. For these consumers, their own personal taste, the convenience and time savings associated with consuming UPFs, coupled with the affordability of this product, encourage UPFs consumption [33,34].

The nutrient contents of UPFs were found to be generally lower than that of MPFs among adults in Guangdong Province. However, UPFs exhibited higher carbohydrate energy ratios and sodium content than MPFs. The evidence from several countries and regions consistently demonstrated that UPFs were less nutritious than unprocessed foods [4,35,36]. As the consumption of UPFs rises, there is a notable decline in the intake of certain nutrients, suggesting that increased consumption of UPFs may contribute to a decline in nutritional quality. A recent meta-analysis of nutritional data from nationally representative samples from around the world revealed a negative correlation between increased UPFs consumption and the nutritional quality of the diet [37].

While UPFs typically contain a higher percentage of carbohydrates, our findings indicate a reduction in the proportion of carbohydrate energy supply in higher grades of UPFs intake compared to lower intake grades. This may be attributed to the observed shift in energy sources from carbohydrates to fat. The consumption of UPFs is associated with a higher energy density and sodium content. The observed trend of declining sodium nutrient density concomitant with the rising energy share of UPFs may be attributed to the fact that sodium intake is primarily derived from flavorings such as sauces, soy sauces, and chicken essence, which have a markedly high sodium content but offer minimal energy [38,39,40], which results in a higher sodium nutrient density in the lower energy share. Nevertheless, in general, the intake of these foods may contribute to an excess of energy intake, which in turn may lead to an accumulation of excess body fat and the development of obesity [41,42]. In addition to the potential for poor nutritional status, the processing of food may also result in structural and compositional alterations, which could subsequently lead to adverse health outcomes [43,44].

Since UPFs lack balance in their nutrient content and are characterized by the presence of numerous additives and artificial flavorings, numerous studies have explored the potential association between their consumption and subsequent health outcomes. A number of epidemiological surveys and clinical studies have demonstrated a robust correlation between the consumption of UPFs and an array of chronic diseases, including cardiovascular disease, type 2 diabetes, and cancer [9,45,46]. For example, a large prospective cohort study found that a 10% increase in daily intake of UPFs was associated with a 6% increase in risk of overall cancer death [47]. Furthermore, the impact of UPFs on children’s health is significant, contributing not only to childhood obesity but also potentially affecting normal growth and neurocognitive development [48,49].

Furthermore, research has evidenced that food additives frequently incorporated into UPFs (including emulsifiers, sweeteners, colors, particles and nanoparticles) exert an impact on the gut, including on the microbiota, gut permeability and gut inflammation [50]. Although the incorporation of an excess of UPFs into the diet will tend to result in a decline in dietary quality; however, there are different findings [51]. This suggests that the NOVA classification system may also be limited in its scope. For example, some nutrient-enhanced foods are defined as UPFs, while some relatively unhealthy foods are placed in the MPFs [52,53]. These findings indicate that the extent of food processing should be incorporated as a factor in future dietary recommendations, alongside nutrients and food groups. From a public health standpoint, it has become crucial to develop guidance on the consumption of UPFs and promote a healthy and balanced diet. Advocating transparency in food labeling, strengthening public health education and formulating relevant policies to regulate the food industry are essential strategies for enhancing public health.

Notable strengths of the study include the use of data from a large sample with provincial-level representation within national data, thus ensuring high external validity. Additionally, through the weighing of food and condiments in a family’s inventory, as recorded in a previously validated continuous three-day 24 h recall, more reliable measurements of various food types were obtained.

When interpreting the current research findings, it is important to consider the limitations of the study. Firstly, the intake of UPFs may be underestimated because, during survey collections, people tend to split the foods they eat, such as breaking down a beef-flavored instant rice into fresh rice and unprocessed beef, leading to the classification of what should be UPF consumption into unprocessed food categories. Secondly, the data over 20 years are not continuous, preventing a more detailed observation of changes over this period. Consequently, additional prospective cohort studies are needed to investigate trends in UPFs consumption.

## 5. Conclusions

The current findings indicated that the consumption of UPFs can be found among all demographic groups and that the intake of UPFs has continued to increase over the past two decades for the majority of individuals. In light of the rising consumption of UPFs and the growing body of evidence suggesting a link between these products and chronic diseases, it is crucial to encourage healthier food choices and a more balanced diet through effective nutrition education initiatives to prevent the potential health risks associated with such dietary patterns.

## Figures and Tables

**Table 1:** Demographic and basic characteristics of participants in each CNNS survey.

Characteristic	Number of Participants (Weighted %) in CNNS Surveys ^1^		
	2002	2012	2022
**Gender**			
Male	2517 (55.2)	1759 (55.0)	1401 (56.4)
Female	2775 (44.8)	2169 (45.0)	1598 (43.6)
**Place of residence**			
urban city	2538 (75.9)	2726 (76.5)	2185 (72.3)
rural areas	2754 (24.1)	1202 (23.5)	814 (27.7)
**Age group**			
18–44 y	2576 (54.1)	1922 (56.3)	1110 (46.9)
45–59 y	1542 (26.4)	1157 (26.1)	930 (31.2)
60+ y	1174 (19.5)	849 (17.7)	959 (22.0)
**Education level**			
Primary school degree or below	1944 (35.2)	1058 (19.8)	860 (20.0)
Secondary school degree	2782 (54.4)	2096 (53.4)	1412 (46.8)
College degree or above	566 (10.4)	774 (26.8)	727 (33.2)
**Occupation**			
Student	74 (2.0)	50 (1.8)	17 (1.2)
Retired or unemployed	1911 (39.3)	1404 (31.7)	1174 (29.8)
Office worker	912 (20.2)	822 (27.7)	443 (19.9)
Business service	477 (12.1)	585 (17.5)	274 (9.7)
Manual labor	1742 (19.7)	662 (12.1)	353 (9.9)
Others	176 (6.7)	405 (9.3)	738 (29.4)
**Income level ^2^**			
Poverty	2954 (42.0)	862 (15.4)	1251 (37.4)
Non-poverty	2148 (52.8)	1616 (39.0)	1697 (61.1)
No response	190 (5.2)	1450 (45.7)	51 (1.6)

^1^ Weights adjusted for gender, resident and age. ^2^ The year-specific poverty line is set at 50 per cent of the median disposable income per capita for the year, which was RMB 4751 in 2002, RMB 11,759 in 2012 and RMB 20,419 in 2022.

**Table 2:** Change in calculated percentage of energy from NOVA food groups for Guangdong adults by CNNS survey, median (IQR).

NOVA Food Groups	Median (IQR) Percentage of Energy from Food Consumption by CNNS Survey			
	2002	2012	2022	*p*
Minimally processed foods	82.95 (12.36)	72.89 (16.45)	68.69 (23.43)	<0.001
Processed culinary ingredients	13.93 (10.88)	13.17 (10.07)	13.62 (15.93)	<0.001
Processed foods	2.64 (3.5)	7.72 (9.43)	8.87 (11.37)	<0.001
Ultra-processed foods	0.88 (2.54)	6.22 (13.32)	8.52 (18.03)	<0.001

**Table 3:** Change in calculated percentage of energy from UPFs among population subgroups of Guangdong adults by CNNS survey, median (IQR).

Population Groups and Subgroups	Median (IQR) Percentage of Energy from UPFs Consumption by CNNS Survey			
	2002	2012	2022	*p*
**Sex**				
Males	0.9 (2.84)	5.76 (12.51)	8.07 (16.89)	<0.001
Females	0.88 (2.21)	6.6 (13.94)	9.34 (20.42)	<0.001
**Place of residence**				
urban city	1.05 (3.37)	7.8 (14.49)	10.17 (20.06)	<0.001
rural areas	0.46 (0.88)	1.84 (6.94)	3.16 (11.17)	<0.001
**Age group**				
18–44 y	0.93 (2.89)	6.82 (14.19)	9.45 (19.95)	<0.001
45–59 y	0.79 (2.1)	5.76 (12.76)	7.69 (16.68)	<0.001
60+ y	0.85 (2.26)	4.62 (11.81)	7.87 (17.03)	<0.001
**Education level**				
Primary school degree or below	0.95 (2.07)	3.97 (10.45)	5.45 (14.26)	<0.001
Secondary school degree	0.85 (2.77)	5.15 (12.01)	8.3 (17.35)	<0.001
College degree or above	0.81 (2.87)	10.13 (15.08)	11.37 (21.27)	<0.001
**Occupation**				
Student	0.42 (2.46)	6.93 (22.06)	17.26 (29.9)	<0.001
Retired or unemployed	0.95 (2.29)	6.07 (12.19)	7.87 (17.69)	<0.001
Office worker	1.04 (3.72)	8.31 (14.41)	10.22 (20.51)	<0.001
Business service	0.83 (3.3)	5.28 (13.79)	8.34 (15.3)	<0.001
Manual labor	0.66 (1.63)	2.51 (8.31)	5.15 (16.06)	<0.001
Others	0.94 (2.68)	7.41 (14.73)	8.56 (17.59)	<0.001
**Income level**				
Poverty	0.69 (1.59)	4.16 (11.42)	5.55 (13.81)	<0.001
Non-poverty	1.05 (3.5)	6.07 (13.56)	10.22 (20.86)	<0.001
No response	1.02 (2.37)	6.98 (13.57)	5.39 (21.54)	<0.001

**Table 4:** Nutrient profiles of MPFs and UPFs consumed by Guangdong adults in the CNNS 2022 survey, median (IQR).

Nutrients	MPFs	UPFs	*p*
Carbohydrates, % of energy	43.83 (27.95)	56.56 (24.13)	<0.001
Protein, % of energy	22.23 (10.37)	18.36 (13.47)	<0.001
Total fats, % of energy	33.67 (22.97)	25.08 (32.58)	<0.001
Insoluble fiber, g/100 kcal	0.3 (0.38)	0.1 (0.31)	<0.001
Cholesterol, mg/100 kcal	35.25 (29.85)	0.27 (5.53)	<0.001
Vitamin A, μgRAE/100 kcal	37.04 (41.14)	0 (7.93)	<0.001
VitaminC, mg/100 kcal	6.7 (7.81)	0 (0)	<0.001
VitaminE, mg/100 kcal	0.43 (0.37)	0.28 (0.59)	<0.001
Carotene, μg/100 kcal	130.45 (214.7)	0 (0)	<0.001
Calcium, mg/100 kcal	27.74 (22.21)	21.36 (43.17)	<0.001
Potassium, mg/100 kcal	161.58 (124.37)	106.92 (370.04)	<0.001
Sodium, mg/100 kcal	29.31 (20.58)	749.33 (4324.66)	<0.001
Iron, mg/100 kcal	1.09 (0.56)	1.52 (3.46)	<0.001
Selenium, μg/100 kcal	3.53 (2.13)	2.16 (1.85)	<0.001
Phosphorus, mg/100 kcal	65.78 (27.29)	52.16 (70.59)	<0.001
Magnesium, mg/100 kcal	16.04 (7.67)	19.01 (68.29)	<0.001
Zinc, mg/100 kcal	0.76 (0.3)	0.61 (1.02)	<0.001

**Table 5:** Nutrient density of UPFs consumption quartiles of adults in Guangdong province (2022), median (IQR).

Nutrients	Q1	Q2	Q3	Q4	*p*
Carbohydrates, % of energy	59.7 (32.46)	58.25 (35.8)	52.43 (34.45)	47.98 (29.5)	0.008
Protein, % of energy	36.29 (18.12)	19.1 (11.59)	13.62 (6.33)	14.11 (6.12)	<0.001
Total fats, % of energy	3.48 (3.31)	22.58 (33.1)	33.93 (30.2)	37.91 (24)	<0.001
Insoluble fiber, g/100 kcal	0.07 (0.32)	0.09 (0.27)	0.11 (0.21)	0.11 (0.35)	0.008
Cholesterol, mg/100 kcal	0 (0)	0.09 (9.73)	2.28 (8.22)	3.1 (8.76)	<0.001
Vitamin A, μgRAE /100 kcal	0 (0)	0 (7.36)	1.73 (11.18)	5.27 (13.77)	<0.001
Vitamin C, mg/100 kcal	0 (0)	0 (0)	0 (0)	0 (0.05)	<0.001
Vitamin E, mg/100 kcal	0 (0)	0.3 (0.71)	0.44 (0.41)	0.46 (0.5)	<0.001
Carotene, μg/100 kcal	0 (0)	0 (0)	0 (2.89)	0 (5.69)	<0.001
Calcium, mg/100 kcal	80 (62.51)	20.34 (31.02)	15.05 (17.22)	13.3 (14.3)	<0.001
Potassium, mg/100 kcal	566.95 (694.62)	128.55 (141.86)	79.28 (61.44)	67.48 (41.93)	<0.001
Sodium, mg/100 kcal	9138.1 (7019.84)	1237.04 (1917.36)	437.01 (489.43)	264.75 (244.7)	<0.001
Iron, mg/100 kcal	12.07 (8.2)	1.78 (2.11)	1.03 (0.88)	0.82 (0.69)	<0.001
Selenium, μg/100 kcal	2.93 (4.29)	1.96 (1.9)	1.93 (1.42)	1.76 (1.33)	<0.001
Phosphorus, mg/100 kcal	262.12 (198.04)	52.67 (51.44)	40.4 (26.63)	37.92 (20.44)	<0.001
Magnesium, mg/100 kcal	147.11 (128.57)	23.22 (39.54)	12.83 (12.01)	9.62 (9.38)	<0.001
Zinc, mg/100 kcal	1.86 (0.76)	0.59 (0.53)	0.42 (0.41)	0.43 (0.36)	<0.001

## Data Availability

The original contributions presented in the study are included in the article, further inquiries can be directed to the corresponding author. The data are not publicly available according to the National Institute for Nutrition and Health and the Chinese Center for Disease Control and Prevention.

## Supplementary Materials

The following supporting information can be downloaded at: https://www.mdpi.com/article/10.3390/nu16234008/s1, Table S1. Number of NOVA Subgroup Foods Consumers and the Percentage of Total Population for Guangdong Adults by CNNS Survey. Table S2. Change in Calculated Percentage of Energy from NOVA Food Subgroups Consumers for Guangdong Adults by CNNS Survey, median (IQR).
