# Supplementary material for: Trends in Consumption of Ultra-Processed Foods Among Adults in Southern China: Analysis of Serial Cross-Sectional Health Survey Data 2002–2022
Source: Nutrients. 2024 Nov 23;16(23):4008. doi: 10.3390/nu16234008 (PMC11643392; doi:10.3390/nu16234008)
Supplement: Supplementary file 1 [file nutrients-16-04008-s001.zip › nutrients-3312212-supplementary.pdf]

Table S1. Number of NOVA Subgroup Foods Consumers and the Percentage of Total Population for Guangdong Adults by CNNS Survey.

**NOVA food groups and subgroups**		**Number of participants(%) in CNNS surveys**		
		**2002**	**2012**	**2022**
**Minimally processed foods**	cereal	5292(100)	3926(99.95)	2985(99.53)
	nuts,seeds,and legumes	2948(55.71)	2139(54.46)	1094(36.48)
	vegetables	5218(98.6)	3924(99.9)	3015(100.53)
	fruits	2548(48.15)	1975(50.28)	952(31.74)
	meats,poultry,fish and eggs	5192(98.11)	3917(99.72)	3028(100.97)
	milk and yogurt	641(12.11)	790(20.11)	707(23.57)
	other	0(0)	533(13.57)	706(23.54)
**Processed culinary**	animal fats	1056(19.95)	691(17.59)	59(1.97)
	plant oils	4527(85.54)	3763(95.8)	2860(95.37)
	sugar and starch	2530(47.81)	1953(49.72)	878(29.28)
**Processed foods**	cereal products	0(0)	2150(54.74)	1949(64.99)
	canned fruits	0(0)	293(7.46)	14(0.47)
	milk powder and cheese	2(0.04)	64(1.63)	88(2.93)
	canned, smoked or pickled meats and fish	339(6.41)	295(7.51)	39(1.3)
	other	291(5.5)	325(8.27)	221(7.37)
**Ultra-processed foods**	industrial cereal foods	134(2.53)	1048(26.68)	705(23.51)
	prepared dishes	559(10.56)	1545(39.33)	1587(52.92)
	snacks and sweets	232(4.38)	560(14.26)	520(17.34)

	sugar-sweetened beverages	270(5.1)	268(6.82)	230(7.67)
	flavored dairy products and dairy substitutes	104(1.97)	75(1.91)	50(1.67)
	other	4789(90.5)	3833(97.58)	2893(96.47)

Table S2. Change in Calculated Percentage of Energy from NOVA Food Subgroups Consumers for Guangdong Adults by CNNS Survey, median (IQR).

**NOVA food groups and subgroups**		**Median (IQR) percentage of energy from food consumption by CNNS Survey**			
		**2002**	**2012**	**2022**	***p***
**Minimally processed foods**	cereal	49.68(20.22)	31.77(19.46)	21.18(25.28)	＜0.001
	nuts,seeds,and legumes	2.91(4.78)	2.54(3.68)	0.96(2.42)	＜0.001
	vegetables	3.28(3.06)	4.54(3.34)	2.98(3.2)	＜0.001
	fruits	2.14(2.56)	3.09(3.55)	1.94(2.48)	＜0.001
	meats,poultry,fish and eggs	22(14.02)	25.5(13.73)	29.8(21.95)	＜0.001
	milk and yogurt	2.21(2.12)	3.42(2.83)	3.26(3.19)	＜0.001
	other	0 (0)	0(0.08)	0.11(0.84)	＜0.001
**Processed culinary**	animal fats	12.34(12.34)	5.35(7.08)	2.19(13.83)	＜0.001
	plant oils	12.73(10.24)	12.08(10.49)	13.32(15.5)	＜0.001
	sugar and starch	0.84(1.57)	0.65(0.81)	0.78(1.19)	＜0.001
**Processed foods**	cereal products	0 (0)	8.22(8.68)	8.82(10.51)	＜0.001
	canned fruits	0 (0)	0.16(0.21)	0.73(0.57)	＜0.001

	milk powder and cheese	10.07(2.4)	4.33(6.24)	18.97(27.56)	＜0.001
	canned, smoked or pickled meats and fish	3.11(3.44)	2.18(2.93)	1.87(2.16)	＜0.001
	other	1.32(3.5)	2.89(4.9)	0.94(2.07)	＜0.001
**Ultra-processed foods**	industrial cereal foods	0.53(1.4)	6.39(6.57)	7.15(8.14)	＜0.001
	prepared dishes	3.47(3.63)	7.62(8.58)	10.38(13.69)	＜0.001
	snacks and sweets	7.11(10.82)	6.26(6.82)	6.01(7.61)	0.47
	sugar-sweetened beverages	1.45(2.69)	2.49(2.96)	1.94(2.28)	＜0.001
	flavored dairy products and dairy substitutes	4.5(6.95)	2.81(3.17)	5.34(23.48)	＜0.001
	other	0.54(0.66)	0.5(0.77)	0.63(1.35)	＜0.001
